# Height, shape and anterior-posterior diameter of pituitary gland on magnetic resonance imaging among patients with multiple sclerosis compared to normal individuals

**Published:** 2017-10-07

**Authors:** Mohammad Saba, Hossein Ali Ebrahimi, Habibeh Ahmadi-Pour, Mohammad Khodadoust

**Affiliations:** 1 Department of Radiology, Afzalipour School of Medicine, Kerman University of Medical Sciences, Kerman, Iran; 2 Neurology Research Center, Kerman University of Medical Sciences, Kerman, Iran; 3 Department of Community Medicine, Afzalipour School of Medicine, Kerman University of Medical Sciences, Kerman, Iran

**Keywords:** Multiple Sclerosis, Pituitary, Height, Shape, Magnetic Resonance Imaging

## Abstract

**Background:** Several studies indicate contribution of hypothalamus-pituitary-adrenal (HPA) axis in multiple sclerosis (MS) disease. This study was designed to determine whether there is an effective difference in pituitary height, shape, and anterior-posterior diameter (APD) between patients with MS and the control group.

**Methods:** In this study, sagittal pituitary height and APD of 134 men and women (64 patients with MS and 70 healthy subjects as control group) were measured by T1 sequence magnetic resonance imaging (MRI). All the subjects were free of sellar or parasellar pathology without a history of surgical intervention or prolactin affecting drugs like bromocriptine and cabergoline or corticosteroid consumption.

**Results:** Mean height of pituitary gland was 6.62 ± 1.43 and 5.78 ± 1.15 mm for patients and the control group, respectively, and the difference between the two groups was statistically significant (P = 0.001). Mean APD was 10.40 ± 1.29 mm for the group of patients and 10.25 ± 1.41 mm for the control group, respectively, without significant differences. 46.9%, 37.5%, and 15.6% of patients had flat, convex, and concave hypophyseal surfaces, respectively. This rate was 50%, 30%, and 20% among the control group, respectively. There was no significant difference between our measurements among patients on whom imaging study was performed at time of disease onset with others.

**Conclusion:** Mean height of pituitary gland among patients with MS was significantly greater than the control group (P = 0.001). So can we consider the same etiology for pituitary hypertrophy among patients with MS as a hypothesis?

## Introduction

Multiple Sclerosis (MS) is known as the most common demyelinating disease of human central nervous system. It’s a complex neurological condition with demyelination and axonal loss.^1^^,^^2^

MS incidence rate has been estimated as 2.5 million individuals worldwide. The incidence of MS in Iran is estimated to be 54.51 per 100000 population,^3^ and 31.5 individuals per 100000 in Kerman Province, Iran.^4^

The stress system has peripheral and central parts: the hypothalamus-pituitary-adrenal (HPA) axis, and the sympathetic and adrenomedullary systems are the peripheral limbs, however, central components are in the hypothalamus and the brain stem; the main function of this system is to maintain basal and stress-related homeostasis.^5^

Several studies indicate the contribution of HPA axis among patients with MS, increasing activity of HPA.^6^ This study was designed in order to evaluate these responses on anatomy of pituitary gland and to determine whether there are differences in height, shape and anterior-posterior diameter (APD) of pituitary between patients with MS and the control group.

## Materials and Methods

In this case-control study, 64 definite patients with MS (57 women and 7 men) with the age range of 15-40 years old and 70 healthy, age and sex–matched controls were selected according to revised Mc-Donald's criteria. All the patients and control group had no history of sellar or parasellar pathology or cranial surgical intervention. During last 3 months prior to study, they had no history of consumption of drugs like bromocriptine, corticosteroids, and cabergoline, which may affect HPA.

Pregnancy and lactation were considered as criteria of excluding from the study. Imaging was performed using GE Signa Excite, 1.5 Tesla Scanner.

For 28 patients (43.75%), imaging was performed at disease onset (before MS treatment).

## Results

The mean age was 27.06 and 27.54 years for patients and the control group, respectively, without statistically significant difference (P = 0.580).

The mean height of pituitary gland was respectively 6.62 ± 1.43 and 5.78 ± 1.15 mm among patients with MS and among the control group, being higher for patients than the control group (P = 0.001).

The mean APD diameter was 10.40 ± 1.29 mm for the group of patients and 10.25 ± 1.40 mm for the control group, respectively (P = 0.520). 

46.9%, 37.5%, and 15.6% of patients had flat, convex, and concave hypophyseal surfaces, respectively. These rates were respectively 50%, 30%, and 20% among the control group. There was no difference in shape distribution percentage between the two groups (P = 0.610). It can be observed that the convex shape has increased among the patients with MS, however the concave shape has decreased among them.

There is no significant difference between the patient individuals and control group members in our measurements (height: 6.88 ± 1.52, APD: 10.45 ± 1.32) versus (height 6.42 ± 1.34, APD: 10.37 ± 1.12). The imaging study among the patients was performed at the time of disease onset (P < 0.050).

All the patients and controls were divided into 3 groups according to their ages.

The mean height of pituitary gland for men in the group of patients was respectively 6.60 ± 1.66 mm in the age range of 15 to 20 years old, 7.06 ± 1.19 mm in the age range of 21 to 30 years old, and 6.16 ± 1.60 mm in the age range of 31 to 40 years old. There was no significant difference with P value equal to 0.11. Mean AP diameter of hypophysis in above mentioned groups was 9.98 ± 1.30, 9.99 ± 1.36 and 10.37 ± 1.52 mm respectively.

## Discussion

Our results showed higher pituitary gland height among patients with MS compared to healthy controls. The height and AP diameter values of hypophysis among normal individuals in Kerman Province are 5.78 and 10.25 mm, respectively.^7^ These findings are in agreement with other studies.^8^

To our knowledge, we didn’t find any report about measurements of pituitary gland among patients with MS in the researches published in English language.

Clinical and experimental studies show that HPA axis has abnormality among patients with MS undergoing severe levels of MS. It might be associated with the pathogenesis of the disease. Hyperactivity of HPA axis is seen among 50% of patients with MS.^8^

Pituitary height was significantly more among 15-to 20-year-old controls. We know that pituitary height is significantly greater among women than men, especially among the adolescent and young individuals; this problem is known as physiologic hypertrophy.^9^ MS disease is more frequent among women in almost the same age group.

**Table 1:** Mean of height and anterior-posterior diameter (APD) among patients and control group

**Variable**	**Group**	**n**	**Mean ± SD**	**P**
Height (mm)	Patients	64	6.62 ± 1.43	0.001
	Controls	70	5.78 ± 1.15	
APD (mm)	Patients	64	10.40 ± 1.29	0.520
	Controls	70	10.25 ± 1.41	
Age (year)	Patients	64	27.06 ± 5.03	0.580
	Controls	70	27.54 ± 5.07	

SD: Standard deviation; APD: Anterior-posterior diameter

Among individuals undergoing MS with fatigue, HPA axis is more active compared to patients without fatigue. Adrenocorticotropic hormone (ACTH) concentration is significantly high in patients with fatigue. Proinflammatory cytokines are increased among patients with MS, which might be the reason for fatigue and changes of HPA axis.^10^

In this study, 28 cases (43.75%) of patients with MS did not receive interferon-beta (IFN-), however, 36 cases (56.25%) received IFN-. There is no significant difference in height and AP diameter of hypophysis (Table 1). Acute IFN- administration transiently activates the hypothalamic-pituitary-adrenal (HPA) axis, however, long-term treatment reduced the responsiveness of the HPA axis to the injection.^11^

## Conclusion

The mean height of pituitary gland among patients with MS was significantly greater than that of the control group (P = 0.001). So can we consider the same etiology for pituitary hypertrophy among patients with MS as a hypothesis?

## Notes:

**How to cite this article:** Saba M, Ebrahimi HA, Ahmadi-Pour H, Khodadoust M. Height, shape and anterior-posterior diameter of pituitary gland on magnetic resonance imaging among patients with multiple sclerosis compared to normal individuals. Iran J Neurol 2017; 16(4): 218-20.
